# FECAL TRANSPLANT FROM LONG LIVING AMES DWARF MICE ALTERS THE MICROBIOME AND BIOMARKERS OF LIVER HEALTH IN CONTROL MICE

**DOI:** 10.1093/geroni/igae098.3659

**Published:** 2024-12-31

**Authors:** Sarah Ashiqueali, Natalie Hayslip, Diptaraj Chaudhari, Augusto Schneider, Xiang Zhu, Sarah Noureddine, Hariom Yadav, Michal Masternak

**Affiliations:** Mayo Clinic, Rochester, Minnesota, United States; University of Central Florida, Orlando, Florida, United States; Wayne state university, Wayne State University, Michigan, United States; Universidade Federal de Pelotas, Pelotas, Rio Grande do Sul, Brazil; University of Central Florida, Orlando, Florida, United States; Hesperos Inc., Orlando, Florida, United States; University of South Florida, Tampa, Florida, United States; University of Central Florida College of Medicine, Orlando, Florida, United States

## Abstract

Aging is associated with intestinal dysbiosis, a condition characterized by diminished microbial biodiversity and inflammation. This leads to increased vulnerability to extraintestinal manifestations such as autoimmune, metabolic, and neurodegenerative conditions and accelerates mortality. As such, modulation of the gut microbiome is a promising way to extend healthspan. In this study, we explore the effects of fecal microbiota transplant (FMT) from long-living Ames dwarf donors to their normal littermates, and vice versa, on the recipient gut microbiota and liver transcriptome. Importantly, our previous studies highlight differences between the microbiomes of Ames dwarf relative to their normal siblings, potentially contributing to their extended lifespan and healthspan. Our findings demonstrate that FMT from Ames dwarf mice to normal mice significantly alters the recipient’s gut microbiota, reprograms bacterial metabolic functions related to healthy aging, and changes the liver transcriptome, indicating improved metabolic health. Our liver mRNA sequencing and RT-PCR validation reveals that FMT in normal recipient animals administered dwarf donor stool may contribute to the significant downregulation of p21, Elovl3, and Insig2, genes involved with cellular senescence and liver metabolic pathways. Our data suggest a regulatory axis exists between the gut and liver, highlighting the potential of microbiome-targeted therapies in promoting healthy aging. Future research should validate these findings and study the underlying mechanisms that confer geroprotection.

